# Assessing the Feasibility and Acceptability of a Bespoke Large Language Model Pipeline to Extract Data From Different Study Designs for Public Health Evidence Reviews

**DOI:** 10.1002/cesm.70061

**Published:** 2025-11-04

**Authors:** Zalaya Simmons, Beti Evans, Tamsyn Harris, Harry Woolnough, Lauren Dunn, Jonathon Fuller, Kerry Cella, Daphne Duval

**Affiliations:** ^1^ Research, Evidence and Knowledge Division, Chief Scientific Officer Group UK Health Security Agency (UKHSA) London UK; ^2^ Centre for Climate and Health Security Chief Scientific Officer Group, UKHSA London UK; ^3^ All Hazards Public Health Response Division Chief Medical Advisor Group, UKHSA London UK; ^4^ Data Science and Geospatial Division Chief Data Officer Group, UKHSA London UK; ^5^ HM Treasury London UK; ^6^ London Borough of Camden London UK; ^7^ Department for Science Innovation and Technology London UK

**Keywords:** artificial intelligence, data extraction, evidence synthesis, feasibility, large language model, public health, systematic review

## Abstract

**Introduction:**

Data extraction is a critical but resource‐intensive step of the evidence review process. Whilst there is evidence that artificial intelligence (AI) and large language models (LLMs) can improve the efficiency of data extraction from randomized controlled trials, their potential for other study designs is unclear. In this context, this study aimed to evaluate the performance of a bespoke LLM model pipeline (Retrieval‐Augmented Generation pipeline utilizing LLaMa 3‐70B) to automate data extraction from a range of study designs by assessing the accuracy and reliability of the extractions measured as error types and acceptability.

**Methods:**

Accuracy was assessed by retrospectively comparing the LLM extractions against human extractions from a review previously conducted by the authors. A total of 173 data fields from 24 articles (including experimental, observational, qualitative, and modeling studies) were assessed, of which three were used for prompt engineering. Reliability was assessed by calculating the mean maximum agreement rate (the highest proportion of identical returns from 10 consecutive extractions) for 116 data fields from 16 of the 24 studies. An evaluation framework was developed to assess the accuracy and reliability of LLM outputs measured as error types and acceptability (acceptability was assessed on whether it would be usable in real‐world settings if the model acted as one reviewer and a human as a second reviewer).

**Results:**

Of the 173 data fields evaluated for accuracy, 68% were rated by human reviewers as acceptable (consistent with what is deemed to be acceptable data extraction from a human reviewer). However, acceptability ratings varied depending on the data field extracted (33% to 100%), with at least 90% acceptability for “objective,” “setting,” and “study design,” but 54% or less for data fields such as “outcome” and “time period.” For reliability, the mean maximum agreement rate was 0.71 (SD: 0.28), with variation across different data fields.

**Conclusion:**

This evaluation demonstrates the potential for LLMs, when paired with human quality assurance, to support data extraction in evidence reviews that include a range of study designs. However, further improvements in performance and validation are required before the model can be introduced into review workflows.

## 1 Introduction

The advancement of artificial intelligence (AI) offers new opportunities to improve the efficiency and scalability of evidence reviews by automating steps in the review process [1]. Traditional machine learning approaches, which involve training algorithms on structured and task‐specific data sets, can already be integrated into the evidence review process, such as the use of machine learning algorithms to prioritize relevant articles in the title and abstract screening process [2, 3].

The emergence of generative large language models (LLMs), such as GPT, Claude, and LLaMa, has introduced further opportunities for automation in the evidence review process. LLMs are pretrained on large quantities of unstructured text which enables them to follow instructions in natural language and be applied more flexibly across a wider range of tasks [4], including extracting textual and numerical data from full‐text articles [5] or drafting reports [6].

LLMs have shown promising performance for some steps of the evidence review process, such as screening [7] and data extraction [8, 9, 10, 11]. However, results have been less promising for other steps, such as search strategy generation [12] or risk of bias assessments [13]. Additional limitations, including the hallucination, misrepresentation, or oversimplification of data, raise concerns around transparency, reproducibility, validity, and integrity of outputs produced by LLMs [1].

Within the evidence review process, data extraction can be particularly resource and time‐intensive, and requires a high degree of accuracy to uphold validity [14]. There is the potential to use LLMs alongside human reviewers to semi‐automate this task to improve efficiency. However, while LLMs have shown promising results when extracting data from randomized controlled trials (RCTs) [8, 9, 10, 15], their ability to extract data from a wider range of study designs, including observational research, is unclear. This is particularly important in public health, where RCTs are not always feasible or ethical and evidence is often synthesized from diverse study designs to inform policy and practice. To address this gap, data scientists and evidence reviewers at UKHSA co‐developed a bespoke LLM pipeline for data extraction from a range of study designs for evidence reviews (evidence synthesis outputs following systematic methodologies), integrated within UKHSA infrastructure and designed to work with human oversight.

The aim of this feasibility study was to evaluate the performance of a bespoke LLM pipeline designed to automate data extraction in evidence reviews and assess the accuracy and reliability of outputs measured as error types and acceptability.

## 2 Methods

**Figure 1:**
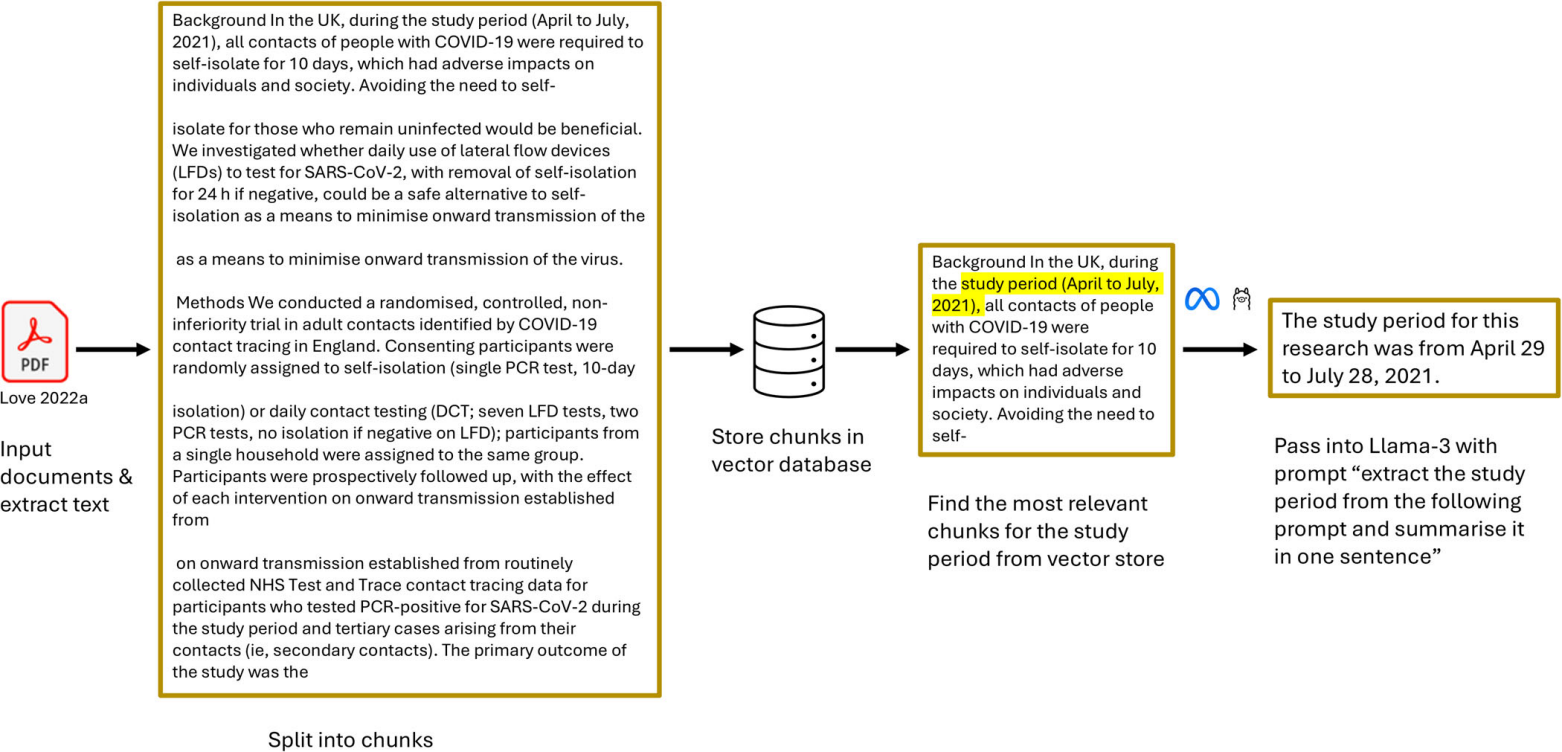
RAG pipeline.

A Retrieval‐Augmented Generation (RAG) pipeline utilizing a LLM was developed to automate data extraction (see Figure 1). The LLM used was LLaMa 3‐70B, which had previously been identified as higher‐performing for classification or data extraction‐related tasks in the public health context [16]. As this LLM is hosted within UKHSA's secure infrastructure, data (including articles' full text) does not leave UKHSA's internal environment, with no data used to train the LLM and no third‐party access permitted.

### 2.1 Reference Standard

LLM data extraction outputs were compared against data that had been extracted and quality assured by two reviewers as part of a published rapid mapping review [17, 18]. The rapid mapping review aimed to identify and categorize the evidence generated during the COVID‐19 pandemic on the effectiveness of nonpharmaceutical interventions (NPIs) implemented in community settings in the UK.

To assess accuracy, a convenience sample of 24 studies was selected from the 151 studies included in the review, ensuring the sample contained examples of the different study designs included (two RCTs, five prospective longitudinal, three cross‐sectional, four ecological, four mixed‐methods, three qualitative, and three modeling studies) [19, 20, 21, 22, 23, 24, 25, 26, 27, 28, 29, 30, 31, 32, 33, 34, 35, 36, 37, 38, 39, 40, 41, 42]. This was to ensure that the model's performance in extracting data from different study designs that are regularly encountered when reviewing evidence relevant to public health could be evaluated.

For reliability, the sample was further reduced to 16 studies (2 RCTs, 3 prospective longitudinal, 1 cross‐sectional, 4 ecological, 2 mixed‐methods, 2 qualitative, and 2 modeling) [19, 20, 21, 23, 24, 25, 26, 27, 28, 29, 30, 33, 35, 36, 38, 41] to make the analysis manageable (each data field was extracted 10 times) whilst maintaining a range of study designs.

### 2.2 Data Fields Extracted

In the review used as a reference standard [17, 18], 10 or 11 data fields were extracted for each study, with the exact number and type of fields depending on the study design: some fields such as “study objective” or “study period” were extracted for all study designs, while other fields were specific (for instance, “scenario” was only extracted for modeling studies).

For this evaluation, the same data fields were extracted, except “intervention” (because of the overlap with “NPI”), “funding,” and “research group.” As a result, seven or eight data fields were extracted for each study (see Table 1 for description of the data fields), resulting in a total of 173 data fields from 24 studies extracted for accuracy and 116 data fields from 16 studies for reliability.

**Table 1:** Data fields extracted and prompts used.

Data field	Study designs for which data field extracted	Data field prompt used in the model	Expected extraction	Example human reviewer extraction
Study objective	All study designs (RCTs, prospective longitudinal, cross‐sectional, ecological, mixed‐methods, qualitative, modeling)	“What was the objective of the study?”	Summary of the study's objective	To assess whether daily testing of COVID‐19 contacts in secondary schools resulted in a similar control of COVID‐19 transmission and increased school attendance compared to self‐isolating school‐based COVID‐19 contacts
Study design	All study designs except modeling studies	“What was the study design?”	Named study design	Open‐label, cluster‐randomized controlled trial
Setting	All study designs	“What was the setting that the study was set in? (country, city, or specific location)”	Setting such as schools, community‐wide, and/or location/nation if applicable	England (secondary schools and further education colleges)
Participants/population^a^	All study designs except modeling studies	“What were the number of participants and groups used in the study?”	Participant numbers and relevant demographics For ecological studies, details of the population included in the study	Control group: *n* = 99 schools (*n* = 102,859 students, *n* = 11,798 staff); students: 47.1% aged 11–14 years, 48.1% aged 15–18 years Intervention group: *n* = 102 schools (*n* = 111,693 students, *n* = 12,229 staff); students: 45.1% aged 11–14 years, 46.7% aged 15–18 years No restriction on age: students aged 19 years or more attended further education colleges (< 0.1% in both the control and intervention group)
Study period	All study designs	“What was the duration of the study? (e.g., 10 weeks)”	Time period (dates)	10‐week study starting between 19 April to 10 May 2021, to 27 June 2021
NPI	All study designs	“What are the non‐pharmaceutical interventions this study assessed and how were they used?”	Named NPI(s) evaluated in the study	Test and release strategies
Outcomes	All study designs	“What were the outcomes this study monitored?”	Outcomes evaluated in the study relevant to review question	COVID‐19 transmission (rate of symptomatic polymerase chain reaction [PCR]‐positive infection, controlled for community case rates) Lost time (school or work)
Control group	RCTs	“What was the control group used in the study?”	Details of the control used in experimental studies	Students or staff contacts of lateral flow device or PCR positive COVID‐19 cases self‐isolated for 10 days
Model	Modeling studies	“What was the model used in this study?”	Type of model used	An agent‐based model
Data	Modeling studies	“What was the data used in the modeling study?”	Details of data sources used to populate the model	Synthetic population of 103,000 generated from 2011 UK Census data
Scenarios	Modeling studies	“What were the simulated scenarios used in this study?”	Outline of scenarios used in the model	Simulate the impact on viral spread of various combinations of:
				proportion of contact tracing application (CTA) users in the population levels of testing capacity levels of compliance with self‐isolation on the part of CTA users testing policy

a “Population” for ecological studies and “participants” for other study designs.

### 2.3 Pipeline Development and Prompt Engineering

A RAG pipeline utilizing an LLM (LLaMa 3‐70B) was developed to automate data extraction (Figure 1). PDF copies of the articles for data extraction were inputted into the pipeline. Text from each PDF was tokenized (broken down into smaller units [tokens]), split into chunks, and stored in a vector database (a type of database that stores text as numbers based on meaning, so similar pieces of text can be found quickly) using Python and the Langchain framework. The chunking of data is necessary to enable processing within the context window, which equates to the maximum amount of data that can be passed to the LLM at one time. These chunks of text are then turned into numbers (called vectors) that represent their meaning so that the model can understand and compare them. This permits the database to be queried and select the most relevant chunks to the user's query. The chunks were then selected for each data field, from which the LLM extracted and summarized the information using prompting. The summarized information was outputted into an Excel file (see example in Supporting Information S1: Table 1).

Prompts for each data field extracted were developed in an iterative and collaborative process between evidence reviewers and data scientists using four studies [19, 38, 41, 43], three of which were included in the evaluation [19, 38, 41]. To generate consistent responses with a rich vocabulary, the temperature setting of the model (which controls randomness) was set very low (0.01) to reduce randomness and ensure consistency, and the top_p setting (which controls how much of the vocabulary was considered) was set high (0.99). Table 1 shows the prompts used in the LLM and examples of expected extracted content for each data field. See Supporting Information S1: Table 2 for system message and prompt for the overall task.

### 2.4 Evaluation

An evaluation framework was developed to assess the accuracy and reliability of the LLM outputs for each data field measured as error types and acceptability. The framework for error types was based on an evaluation by Gartlehner et al. [8]. Acceptability was incorporated into the evaluation framework to assess whether the output was usable: errors could be acceptable if they were consistent with the types of errors a human reviewer might reasonably make without affecting the interpretation of the study characteristics or findings (such as minor differences in wording). Errors were considered unacceptable if they risked misinterpretation of the study characteristics or findings.

Using this evaluation framework, two reviewers independently assessed the outputs for each data field according to the predefined error and acceptability criteria for accuracy (Table 2) and reliability (Table 3). Assessments were then agreed by consensus, with a third reviewer present to resolve disagreements and record decisions. Reviewers also recorded qualitative observations about the outputs.

**Table 2:** Accuracy evaluation framework.

Error	Description	Acceptability criteria
Accurate extraction	The model returns the correct answer	Extraction *acceptable*
Major error	The data are completely misrepresented in a way that significantly impairs the integrity of the review, for example, including interventions or outcomes that were not evaluated in the article	All major errors are *unacceptable*
Minor error	Involves slight misrepresentation of data that, while inaccurate, does not impact the review's results. This also includes cases where small but noncritical data are missing. For example, for a study conducted in Glasgow, Scotland, the model response is that the study was conducted in a city in Scotland	Minor errors are *acceptable* if they would be an acceptable mistake a human would make, for example, misspelling a word or missing off a small part of a phrase
		*Unacceptable* errors are where a human would be unlikely to make that mistake, or data are misrepresented enough there is potential for it to be misinterpreted
Missing data	The model has not extracted data (whether value not returned, or declared that there was not relevant data in the chunks)	All missing data are *unacceptable* if there is data available in the article and a human reviewer would have been able to extract
Not stated in article	The information of interest has not been stated in the source article (e.g., a study design classified as ecological by a human reviewer but “ecological” is not stated in the source article)	*Acceptable* if the model has been able to infer the correct response, or correctly state that the data were not available in the article
		*Unacceptable* if the model has presented incorrect data from another part of the article
Hallucination	The model has invented data that is not from anywhere in the source article	Hallucination is always *unacceptable*

**Table 3:** Reliability evaluation framework.

Error	Description	Acceptability criteria
Reliable extraction	The model presents the same information for all returns (regardless of accuracy)	Errors are *unacceptable* if variations in data extractions lead to fundamentally different understandings or interpretations of the data. All “value not returned” errors are *unacceptable*. Errors are *acceptable* if, across 10 extractions, the information conveyed is essentially the same, allowing for consistent interpretation despite minor variations in wording or formatting.
Value error	The model presents different information (values) across the 10 returns. For example:	
	There were 10 male participants (1 return) There were 11 male participants (1 return) There were 10 participants (8 returns)	
Voice error	The model presents the same information (values) in different formats across multiple returns (e.g., for every 10 returns, 6 are returned as paragraphs and 4 are returned as bullet points) or has minor variation in wording (e.g., change in tense such as “The number of participants and groups used in the study were…” or “The number of participants and groups used in the study are…”)	
Value not returned	The model has not extracted data	

#### 2.4.1 Accuracy

Accuracy of the LLM outputs for each data field extracted for the 24 studies was assessed by comparing them to the data extracted in the published review [17, 18]. Each data field was assigned one error type: accurate extraction, major error, minor error, missing data, not stated in article, or hallucination (Table 2). Accuracy was calculated as the proportion of extractions with no errors. Accuracy of outputs was categorized as acceptable or not acceptable as per the framework described in Table 2.

#### 2.4.2 Reliability

Reliability of LLM outputs was assessed by running 10 extractions consecutively using identical prompts for each data field of the 16 studies. The reference standard for reliability was that the LLM was expected to return identical values in the same format, independently of accuracy. Within the group of 10 extractions, identical extractions were counted to calculate the maximum agreement rate (the highest proportion of identical extractions within the 10).

The group of 10 extractions for each data field was also evaluated against predefined error types: reliable extraction, value error, voice error, or value not returned (Table 3). A data field could be designated as having both a value and voice error. Reliability of outputs was categorized as acceptable or not acceptable according to the framework described in Table 3.

#### 2.4.3 Analysis

Results were analyzed in Python 3.11 using the Pandas library for data wrangling (also called data cleaning or preprocessing) and the Matplotlib and Plotly libraries for visualization. Distribution of error types and the acceptability of identified errors were analyzed for extractions both overall and disaggregated by data field and study design.

## 3 Results

### 3.1 Accuracy

**Figure 2:**
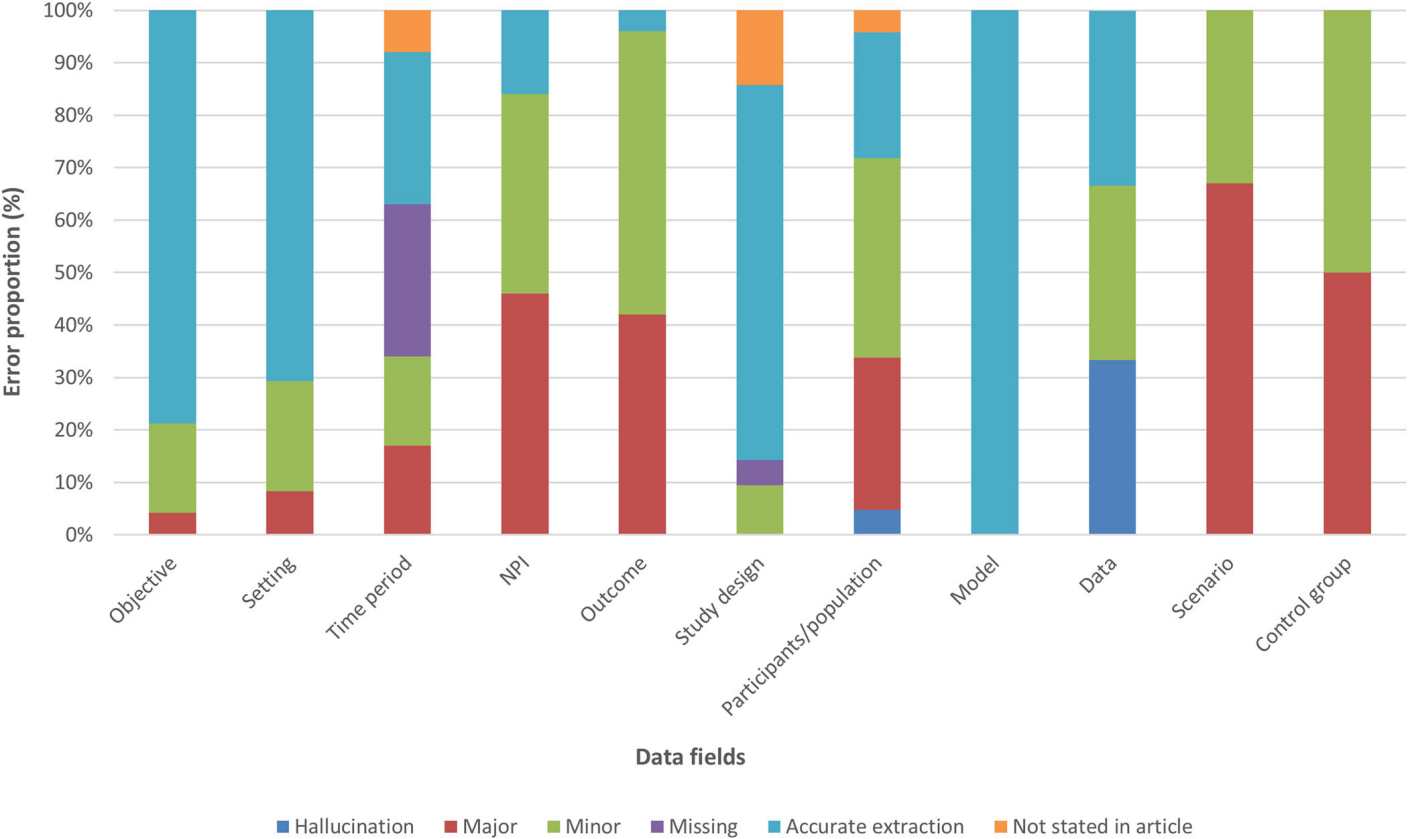
Distribution of accuracy error types by data field.

The findings of accuracy for 173 data fields extracted from the 24 articles are presented in Table 4 and Figure 2. Of the 173 data fields extracted, 42% were accurate, 28% had minor errors, 21% had major errors, 4.6% were categorized as missing data, 3.5% as data not stated in article, and 1.2% of errors were categorized as hallucinations. Overall, the accuracy of 118 of the 173 (68%) data fields was rated as acceptable (consistent with what is deemed to be acceptable data extraction from a human reviewer).

**Table 4:** Accuracy: error types and acceptability by data field (total number of extractions = 173).

Data field (number of studies)	Accurate extraction (%)	Minor error (%)	Major error (%)	Missing (%)	Not stated in article (%)	Hallucination (%)	Acceptability (% acceptable)
Objective (24)	19 (79%)	4 (17%)	1 (4.2%)	0	0	0	22 (92%)
Setting (24)	17 (71%)	5 (21%)	2 (8.3%)	0	0	0	22 (92%)
Time period (24)	7 (29%)	4 (17%)	4 (17%)	7 (29%)	2 (8.3%)	0	13 (54%)
NPI (24)	4 (17%)	9 (38%)	11 (46%)	0	0	0	11 (46%)
Outcome (24)	1 (4.2%)	13 (54%)	10 (42%)	0	0	0	12 (50%)
Study design (21)	15 (71%)	2 (9.5%)	0	1 (4.8%)	3 (14%)	0	19 (90%)
Participants/population (21)	5 (24%)	8 (38%)	6 (29%)	0	1 (4.8%)	1 (4.8%)	14 (67%)
Model (3)	3 (100%)	0	0	0	0	0	3 (100%)
Data (3)	1 (33%)	1 (33%)	0	0	0	1 (33%)	1 (33%)
Scenario (3)	0	1 (33%)	2 (67%)	0	0	0	0
Control group (2)	0	1 (50%)	1 (50%)	0	0	0	1 (50%)
Totals	72 (42%)	48 (28%)	37 (21%)	8 (4.6%)	6 (3.5%)	2 (1.2%)	118 (68%)

However, findings varied across data fields (Table 4). For data fields where at least 21 studies were evaluated, the proportion of data fields with accurate extractions ranged from 4.2% for “outcome” to 79% for “objective,” whilst the proportion with a major error ranged from 0% for “study design” to 46% for “NPI.” Acceptability ranged from 33% to 100%, with acceptability of 90% or higher for data fields “objective,” “setting,” and “study design” (with a high accurate extraction rate of more than 70%) but down to 50% or less for data fields “NPI” and “outcome.” The data field “model” (for modeling studies only) had an accurate extraction rate and acceptability of 100%, however, this was only assessed in three studies.

Accuracy acceptability findings also varied by study design, from 56% overall for RCTs (two studies) to 86% for cross‐sectional studies (three studies) (Supporting Information S1: Table 3). However, the number of studies in each study design category was too low to allow conclusions about differential model performance by study design to be drawn.

### 3.2 Reliability

The reliability of 116 data fields extracted from 16 full‐text articles was assessed, with a mean maximum agreement rate across all data fields of 0.71 (SD: 0.28), showing that on average 7.1 of the 10 consecutive extractions returned the same value for a given data field (Table 5).

**Table 5:** Reliability: maximum agreement rate.

Data field (number of studies)	Maximum agreement rate (mean, SD)
Objective (16)	0.86 (0.23)
Setting (16)	0.86 (0.19)
Time period (16)	0.69 (0.26)
NPI (16)	0.52 (0.25)
Outcome (16)	0.55 (0.26)
Study design (14)	0.94 (0.15)
Participants/population (14)	0.61 (0.22)
Model (2)	1 (0)
Data (2)	0.35 (0.07)
Scenario (2)	0.25 (0.21)
Control group (2)	1 (0)
Overall	0.71 (0.28)

However, the model's reliability varied across different data fields. The data fields “study design,” “setting,” and “objective” showed higher reliability, with mean maximum agreement rates above 0.85. Lower agreement rates were observed for fields such as “outcome” (mean: 0.55) and “participants/population” (mean: 0.61).

**Figure 3:**
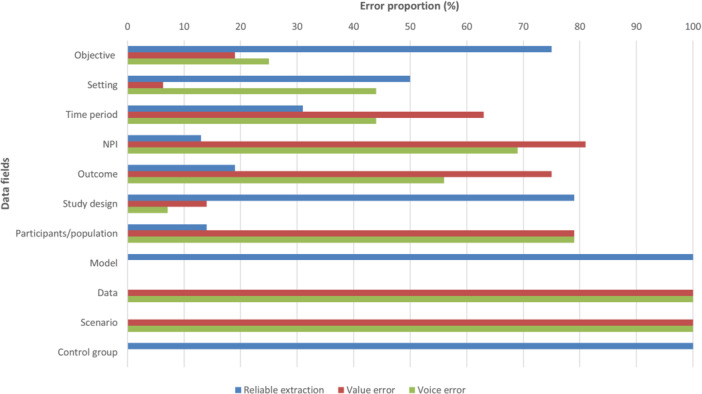
Distribution of reliability error types by data field.

For 47 of the 116 data fields extracted (41%), the 10 extractions were reliable. In the remaining 59% of data fields, unreliability was either due to value errors (differences in the information returned) or voice errors (variation in how the same information was phrased or formatted), or both. Overall, 69% of the reliability error types were designated acceptable (see Figure 3 and Supporting Information S1: Table 4 for details of error types and their acceptability).

### 3.3 Reviewer Observations of the LLM‐Generated Extractions

A common theme in reviewers' observations was that the model tended to be verbose, with broad, unfocused responses which gave the impression of returning all potentially relevant content rather than summarizing relevant information. One reviewer described this as “responding with everything it could find, rather than answering the question.”

Recurring issues were noted across specific data fields, such as “time period,” “outcomes,” and “NPIs”: for “time period,” the model sometimes returned the manuscript submission or acceptance dates, rather than the actual study period; for “outcomes” and “NPIs,” the model sometimes extracted content from the introduction or discussion about broader or future directions, rather than the specific interventions or outcomes assessed in the study, suggesting a potential lack of contextual prioritization.

Reviewers also noted that prompt design appeared to influence the model's behavior and outputs in unintended ways. For example, the prompt for “participant/population” included an instruction to describe any relevant subgroups. In response, the model sometimes inferred subgroups even when none were described in the study.

Although the length and complexity of the outputs may limit its value, reviewers noted the potential benefits of using the model as one reviewer whilst keeping a human in the loop as second reviewer. For instance, in a rapid review, the model could act as a first reviewer and the human as a second reviewer, quality assuring the extraction of the model. In a systematic review context, extraction by both the model and the human would be done independently and then compared to reach consensus.

## 4 Discussion

### 4.1 Main Findings

Accuracy of this bespoke LLM, developed to extract data from a range a study designs, was 42%, whilst a further 28% of outputs had only minor errors. In terms of acceptability, the accuracy of 68% of the outputs was deemed acceptable, meaning that it would be usable in a real‐world evidence review. In terms of reliability, 7 of the 10 consecutive extractions returned, on average, the same value for a given data field.

Accuracy and reliability varied by data field, similar to previous observations [44]. The model demonstrated strong performance for the data fields “study objective,” “study design,” and “setting” where acceptability of accuracy extractions was at least 90%, and reliability had a mean maximum agreement rate of at least 0.86. However, other data fields, such as “outcomes,” “participants/population,” and “time period,” proved more difficult for the model to extract (accuracy 4.2%–29%, accuracy acceptability 50%–64%; mean maximum agreement rates 0.52–0.69), possibly due to varied expression of data across studies and the reporting format (such as in tables) [16].

### 4.2 Findings in the Context of Previous Research

Direct comparison between the findings of this evaluation and other published evaluations of LLM data extraction, which report accuracy of between 68% and 96% [8, 9, 10, 15, 44], is not possible due to differences in the study designs and type of data extracted, and in the LLM and evaluation frameworks used. In particular, published evaluations tend to focus on the extraction of data from RCTs of clinical evidence, therefore mainly numerical data, which follow standardized guidance for reporting [8, 9, 10, 15]. In contrast, this study evaluated the model's performance across a range of study designs assessing public health evidence which tends to be less structured. In this context, it is worth noting that the data extraction for the two RCTs included in this evaluation had the lowest acceptability of all the study designs included, which may reflect the complexity of the interventions and comparisons (both evaluated test and release strategies which involve multistep conditional interventions) [38, 41]. The range of study designs in this evaluation, along with variation in how data are reported across full texts, contributed to an increased complexity of the task but is reflective of the real‐world challenges in evidence reviews in public health.

The results may reflect not only the complexity of the data fields themselves, but also the performance of the pipeline as accuracy can vary depending on the LLM [16, 45] and the effectiveness of prompt engineering [46, 47].

The reliability results, as in other evaluations [44], showed that LLMs can produce different outputs from the same input, which raises an important question around the validity of deriving accuracy from an evaluation of a single run. Setting the temperature close to but not at zero may have resulted in some of the variation seen, although variation has been observed with the temperature set at zero [44].

Criteria used to evaluate accuracy and reliability of LLM extractions differ in published evaluations [8, 9, 10, 15, 44], as do the performance metrics used [48]. The evaluation framework used in this study was composed of a structured assessment of accuracy and reliability error types, and reviewer‐informed acceptability judgment. Evaluating the output in the context of what a human reviewer might reasonably extract by considering acceptability facilitated a more nuanced and practical assessment of model performance than binary accuracy scoring alone. This layered evaluation approach reflects how these tools might be used in real‐world scenarios where human oversight is retained. It also aligns with the potential integration of this pipeline into rapid systematic methodologies, where a human reviewer would quality assure all model‐generated extractions. However, in the absence of standardized metrics and benchmarks for evaluating LLM performance for evidence synthesis applications, interpreting whether performance is “good enough” [48], and whether the LLM should be integrated into the evidence review process remains subjective and context‐specific.

### 4.3 Limitations

Conclusions about differential model performance by study design cannot be drawn due to the small number of studies representing each study design. Three of the 24 studies were used during prompt development, which may have introduced an unknown degree of bias in model performance during evaluation. These studies were retained as part of the build phase's evaluation, which prioritized learning about feasibility and model behavior over a strict separation of the data used to develop the prompts and pipeline from the data used to evaluate the model.

All data fields were weighted equally and did not account for relative differences in complexity or the potential influence of certain data fields on the interpretation of the review findings. A single set of prompts (tailored to studies included in this evaluation) and a single data set were used, so further testing is needed to understand how performance might vary across review types, prompt phrasing, or model configurations.

As this evaluation was completed retrospectively, data were not available to compare the time impact of LLM and human data extraction.

### 4.4 Next Steps

Moving from the build phase to the validation phase, with continued collaboration between data scientists and evidence reviewers, the technical pipeline will be refined and reassessed to improve performance by exploring adjustments to the current RAG‐based approach, and by testing alternative prompting methods that do not rely on retrieval components. Consideration will be given to which type of reviews, study designs, and data fields the pipeline is most appropriate and valuable for. As part of ongoing development, the use of other LLMs within the pipeline is being explored, with the aim to improve accuracy. Next steps will also include assessing the pipeline by conducting a prospective two‐armed evaluation comparing semi‐automated data extraction (LLM extraction checked by a human) to manual data extraction (extraction by a human, checked by a second human) to assess accuracy and reliability, but also impact on time efficiency and workload [48].

In addition, it will be important to consider the principles and practicalities of responsible AI integration and develop appropriate guidelines that promote adherence to legal, ethical, and regulatory requirements, to maintain research quality and integrity [48] within an appropriate governance framework [49]. Users of AI in evidence synthesis must have regard for copyright and intellectual property [11, 48], and ensure transparency in how technical pipelines and the models embedded within them are developed, evaluated, and deployed. Accountability is another important aspect as, when using AI, errors and their impact are the responsibility of the user, and do not transfer to the technology [49].

Finally, establishing minimum thresholds of acceptability for both accuracy and reliability remains a question for future research and is an important consideration for the responsible implementation of AI‐assisted tools in evidence review methods.

These considerations are aligned with recommendations and guidance on responsible AI use in evidence synthesis (RAISE) [48] which highlights the importance of fairness, robustness, and transparency in the application of AI in evidence reviews.

## 5 Conclusion

This evaluation demonstrates the potential for LLMs, when paired with human quality assurance, to support data extraction in evidence reviews that include a range of study designs. However, further improvements in performance are required before the model can be introduced into review workflows. While overall accuracy was modest, the acceptability of outputs, defined as their practical usability by reviewers, was higher, showing potential for real‐world application. Performance varied across data fields, and reliability issues highlighted the limitations of single‐run evaluations. Despite overall variability, the model performed well for the data fields “objective,” “setting,” and “study design.” The evaluation framework, which considered error types and acceptability for accuracy and reliability, offered a more nuanced measure of how these tools might function in evidence review contexts, and the co‐development process between data scientists and evidence reviewers was important in aligning technical design with practical needs. However, further testing across review types, refined prompting strategies, and clearer thresholds for acceptable model performance are needed. Future work must also address the principles of responsible AI integration, including transparency, reproducibility, and appropriate risk‐benefit trade‐offs. With these considerations in place, semi‐automated data extraction using LLMs could play a valuable role in improving the efficiency of public health evidence reviews.

## Author Contributions

**Zalaya Simmons:** conceptualization, methodology, writing – original draft, writing – review and editing, investigation. **Beti Evans:** investigation, writing – original draft, writing – review and editing. **Tamsyn Harris:** investigation, writing – original draft, writing – review and editing. **Harry Woolnough:** methodology, investigation, data curation, writing – review and editing. **Lauren Dunn:** investigation, formal analysis, data curation, writing – review and editing. **Jonathon Fuller:** conceptualization, methodology, writing – review and editing. **Kerry Cella:** data curation, supervision, writing – review and editing. **Daphne Duval:** conceptualization, methodology, writing – review and editing, supervision.

## Disclosure

The views expressed in this article are those of the authors and are not necessarily those of the UK Health Security Agency or the Department of Health and Social Care.

## Conflicts of Interest

K.C., D.D., B.E., T.H., and Z.S. have received free credits (via UKHSA) from Amazon Web Service (AWS) to test how AI solutions offered by AWS may be able to support with text and data extraction. The work presented in the manuscript was not undertaken using AWS, and no contribution was made to the authors or to UKHSA. The other authors declare no conflicts of interest.

## 1 Peer Review

The peer review history for this article is available at https://www.webofscience.com/api/gateway/wos/peer-review/10.1002/cesm.70061.

## Supporting information

LLMs for data extraction in evidence reviews – Supplementary File 1.

LLMs for data extraction in evidence reviews – Supplementary File 2.

LLMs for data extraction in evidence reviews – Supplementary File 3.

## Data Availability

Data analysis files available as Supporting Files 2 and 3.
